# Patient Perspectives on Patronage of Traditional Bone Setting: A Systematic Review

**DOI:** 10.5334/aogh.5295

**Published:** 2026-08-31

**Authors:** Sarah Elizabeth Flynn, Sumner Rajaratnam, Colin Kruse, Daniel K. Kisitu, Bradley A. Petrisor

**Affiliations:** 1 Michael G. DeGroote School of Medicine, McMaster University, Hamilton, Ontario, Canada; 2 Division of Orthopaedic Surgery, McMaster University, Hamilton, Ontario, Canada; 3 Division of Health Research Methodology, McMaster University, Hamilton, Ontario, Canada; 4 Department of Surgery, Mbarara University of Science and Technology, Mbarara, Uganda

**Keywords:** traditional bone setters, bone setting, bone setters

## Abstract

*Introduction:* Traditional bone setters (TBS) treat orthopedic injuries using natural remedies and traditional medicine. These practices can result in complications, including malunion, non-union, or gangrenous extremities. Despite these complications, communities in low- and middle-income countries trust and rely on TBS for musculoskeletal injuries and pathologies. Understanding patients’ motives for patronizing TBS is necessary to work with TBS to reduce complications.

*Objectives:* The objectives of this study were to assess the reasons patients patronize TBS, identify demographic trends associated with TBS usage, and evaluate how patient perspectives inform an understanding of TBS patronage.

*Methods:* Four electronic databases, MEDLINE and Embase (via Ovid), Web of Science, and the Cochrane Central Register of Controlled Trials (CENTRAL), were searched prior to April 2025. Primary studies examining reasons and patient perspectives on visiting TBS were included. Studies not reporting patient perspectives regarding TBS use were excluded. Study quality was assessed using the Joanna Briggs Institute critical appraisal tools. A narrative synthesis was conducted, with pooled descriptive statistics where appropriate.

*Results:* A total of 28 studies involving 8,147 participants were included. A total of 5 articles directly included patients’ perspectives, while 24 articles reported themes among reasons for the patronage of TBS. The main reasons cited for patronizing TBS were the belief in TBS and affordability. Recommendations from family/friends and negative perspectives on allopathic medicine were both frequently cited. No consistent association between participant demographics and TBS patronage was identified.

*Conclusions/Discussion:* The reasons for patronizing TBS are multifaceted, extending beyond pure affordability and accessibility. The analysis showed that the general preference for TBS over allopathic practitioners and the belief in TBS practices are strongly rooted in the individuals and communities that TBS serve. Despite differing recommendations, collaboration with TBS to improve access to care and reduce complication rates was identified as a future direction.

## Introduction

Traditional bone setters (TBS) use natural remedies and traditional practices to treat orthopedic injuries, often with limited allopathic medical training. TBS practices have been in use for centuries, passed down through generations of families that are integrated into the communities that TBS serve [1–4]. TBS practices vary widely, including the use of natural balms, massaging, splinting, or other immobilization techniques to treat orthopedic injuries and conditions, ranging from superficial abrasions to the management of complex fractures [1, 5, 6]. While TBS practices can successfully manage some minor orthopedic fractures, significant complications may occur [7–9]. These complications include fracture malunion, non-union, and gangrenous extremities. These complications can have a dramatic impact on the lives of patients and their families [8, 10–12].

Despite the potentially debilitating complications, patients in low- and middle-income countries (LMICs) continue to trust and use the services of TBS for orthopedic care [5]. This continues despite campaigns to educate patients on the perceived risk of TBS practices [13, 14]. Understanding patients’ reasons and motivations for patronizing TBS is necessary to explore patronage of TBS and to reduce the complications associated with their use. Understanding these reasons may also clarify other cultural, geographic, or financial barriers to accessing allopathic medical care. To our knowledge, this has not been previously investigated or characterized by prior reviews. Thus, a qualitative systematic review of the literature was conducted to assess the reasons and perspectives of patients who patronize TBS.

## Methods

### Study design and overview

This review was designed using the Preferred Reporting Items for Systematic Reviews and Meta-Analyses (PRISMA) guidelines.

### Search strategy and study selection

Four electronic databases, Web of Science, the Cochrane Central Register of Controlled Trials (CENTRAL), MEDLINE, and Embase (both via Ovid), were searched prior to April 2025. A manual search was also conducted. The search terms used were “bone setting,” “bone setters,” and “bonesetters,” and their synonyms. The full search strategy can be found in Appendix A.

Study screening and data extraction were conducted using Covidence systematic review software (Veritas Health Innovation, Melbourne, Australia). Two contributors (SF, SR) independently reviewed the titles, abstracts, and full texts for relevance. Disagreements between reviewers were settled through collaborative discussion with a third reviewer (CK). Study references were also reviewed for any relevant articles.

### Eligibility

This study included articles discussing patients’ reasons for patronizing TBS and exploring their perspectives on TBS. Specifically, articles qualitatively reporting the lived experiences of patients and their perspectives on TBS were included. All articles included were original, peer-reviewed, full-text, and written in the English language.

All studies that did not report TBS patients’ reasoning or perspectives on visiting a TBS were excluded. Articles that were not original, peer-reviewed, full-text, and written in the English language were also excluded.

### Data extraction

Data extraction was completed independently by two reviewers (SF and SR). The data extraction template was created and piloted. The template was validated by contributors SF, SR, and CK, and published on Covidence [15]. The extracted information included study characteristics, patient demographics, study results, including the reasons for attending TBS, and key findings. Given the qualitative nature of the results, no formal statistical analysis was completed in most studies. Instead, descriptive statistics were extracted.

### Methodological quality

Each article was assessed in terms of methodological quality. The Joanna Briggs Institute (JBI) Critical Appraisal tool for qualitative reviews was used for each article [16]. Two reviewers independently performed an overall appraisal, and any disagreements were resolved through discussion and, if necessary, conferring with a third reviewer.

### Data synthesis and analysis

Data management and descriptive analyses were performed using Microsoft Excel (Microsoft Corporation, Redmond, WA, USA). Given the qualitative nature of the articles collected, no quantitative meta-analysis could be conducted. Demographics, including age and sex, were pooled. Articles reporting quantitative survey data were collected in Excel, and where possible, missing values (n, N, %) were calculated.

## Results

### Search results

**Figure 1:**
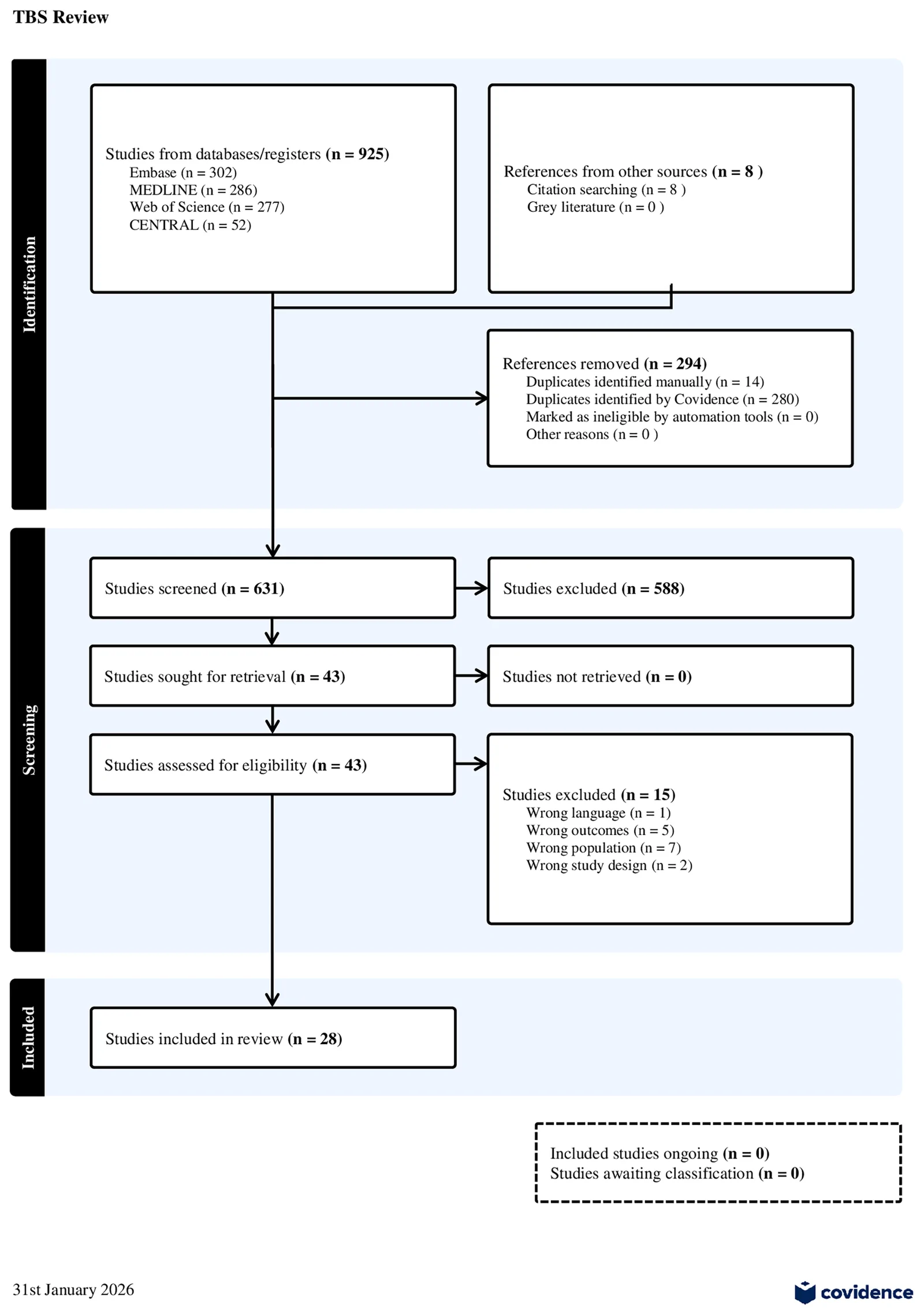
PRISMA diagram.

The search of the 4 electronic databases identified 917 studies that fit the search parameters (Figure 1). Eight additional studies were added following a manual search of databases and article reference lists. Of the 925 studies, 294 duplicates were removed. The 631 remaining articles were then screened based on their title and abstract. A total of 43 studies fit the inclusion criteria, while 588 were excluded. After a full-text review, 15 studies were excluded based on the exclusion criteria of population, outcome, language, or study design. Ultimately, 28 total articles were included in the review.

Of the 28 articles, 5 articles directly quoted patients’ perspectives, while 24 articles reported thematic reasoning behind the patronage of TBS. The results and demographics are summarized in Table 1.

**Table 1:** Study demographics.

AUTHOR, YEAR	LOCATION (REGION, COUNTRY)	STUDY DESIGN	PATIENTS (N)	MEAN AGE (RANGE)	MALE (%)	PRINCIPAL REASON (%)
Abang et al. [10]	Nigeria	Prospective observational	79	36.8 (17–83)	59.5	No choice
Aderibigbe et al. [17]	Nigeria	Cross-sectional	400	36.3	58	Family and friend recommendation
Ali et al. [7]	Pakistan	Retrospective observational	87	16.48 (6–81)	64.4	Availability
Aries et al. [18]	Ghana	Qualitative	29*	–	69	–
Card et al. [19]	Tanzania	Cross-sectional	13**	–	–	Other
Chowdhury et al. [8]	Bangladesh	Prospective observational	150	(1–≥51)	–	Affordability
Dada et al. [9]	Nigeria	Prospective observational	121	29.49 (6 weeks–72)	57	Affordability
Diamond et al. [11]	Nigeria	Cross-sectional	192	35.1 (10 months–76)	64.6	Belief
Edusei et al. [6]	Ghana	Qualitative	16	(16–70)	43.8	–
Golge et al. [12]	Turkey	Cross-sectional	3422	–	58.1	Belief/preference
Hamad et al. [20]	Sudan	Cross-sectional	389	–	53.2	Belief
Hatipoglu and Tatar [1]	Turkey	Qualitative	20	(46–80)	70	Belief + availability
Idris et al. [2]	Sudan	Prospective observational	276	39.3	66	Belief
Jibo et al. [13]	Nigeria	Cross-sectional	224	29 (1–82)	79.9	Affordability
Khan et al. [21]	Pakistan	Prospective observational	60	25.57 (5–65)	73.3	Belief
Kuubiere et al. [22]	Ghana	Observational	80	(7–70)	63.7	Belief
Moton et al. [23]	Pakistan	Cross-sectional	999	32.59 (7–71)	63.2	Belief
Nwachukwu et al. [5]	Nigeria	Qualitative	17	–	–	–
Onyemaechi et al. [24]	Nigeria	Cross-sectional	120	37.4 (0–80)	70	Family and friend recommendation
Panda and Rout [3]	India	Prospective observational	146	48.6	55.5	Other
Ruhinda [25]	Tanzania	Qualitative	103	–	84	–
Safari et al. [14]	Iran	Prospective observational	61	34.04 (11–75)	72.1	Affordability
Sayeed et al. [26]	Uganda	Qualitative	168	–	–	Belief
Solagberu [27]	Nigeria	Prospective observational	295	28 (15–78)	68.1	Affordability
Udosen et al. [4]	Nigeria	Cross-sectional	92	(0–60)	78	Affordability
Yempabe et al. [28]	Ghana	Cross-sectional	64	–	62.5	Affordability
Zarrar et al. [29]	Pakistan	Retrospective observational	362	38.6 (7–87)	61	Affordability
Zehir et al. [30]	Turkey	Cross-sectional	162	27.5	60	Other
**Total**	**–**	**–**	**7394**	**33.4 (SD = 7.7)**	**63.5% (SD = 8.6%)**	

*N = 46 overall, only 29 had previously visited a TBS practitioner.

**N = 212 overall, only 13 had previously visited a TBS practitioner.

### Study demographics

Twenty-eight studies were included in this review: [1–14, 17–30]. The study designs were variable and are reported in Table 1. The studies were conducted in a total of 10 countries. A total of 18 studies were conducted in Africa and 10 in Asia. The country with the most studies was Nigeria with nine studies. Bangladesh, India, Iran, and Uganda had the fewest number of studies, with one each. No study was done in Europe, Australia, North America, or South America.

A total of 8,147 participants were included in the studies. The mean age of participants was 33.4 (SD = 7.7), ranging from 0 to 87. The majority of participants were male (63.5%).

### Study quality

The quality of the studies was variable, ranging from strong to decent, and were all included based on the JBI criteria. Articles of overall low appraisal, as per the JBI critical appraisal tool, were noted [16].

### Patient reported reasoning

The top three patient-reported reasons from each article are reported in Table 2. The number of times each reason was cited is reported in Table 3. Patients cited many different reasons as to why they chose to visit TBS: nine articles cited belief in TBS as the main reason, with two citing a general preference for TBS and belief in TBS skill [2, 3, 11, 12, 20–23, 26]. The reasons for using TBS ranged from spiritual beliefs to confidence in the practices of TBS. Eight articles cited affordability as the main reason [4, 8, 9, 13, 14, 27–29]. Other commonly cited reasons were friends/family recommendations and a negative perspective/fear of allopathic medical institutions, each with two articles citing them as the main reason [17, 19, 24, 30].

**Table 2:** Patient-reported results by article.

AUTHOR, YEAR	PRIMARY REASON (%)	SECONDARY REASONS (%)	TERTIARY REASONS (%)
Abang et al. [10]	No choice	Belief	TBS competency
Aderibigbe et al. [17]	Family and friend recommendation	Availability	Fear of allopathic medicine
Ali et al. [7]	Availability	Family and friend recommendation	Affordability
Card [19]	No improvement at allopathic hospital	Family and friend recommendation/no choice	–
Chowdhury et al. [8]	Affordability	Belief	Quicker service
Dada et al. [9]	Affordability	Family and friend recommendation	Belief in faster healing
Diamond et al. [11]	Belief	Affordability	Availability
Golge et al. [12]	Belief/preference	Fear of disability	Fear of Plaster of Paris
Hamad et al. [20]	Belief	Availability	Family and friend recommendation
Hatipoglu and Tatar [1]	Belief/availability	Affordability/negative perspective of allopathic medicine	–
Idris et al. [2]	Belief	Less time consuming	Affordability
Jibo et al. [13]	Affordability	Avoiding long hospital stay	Belief/family and friend recommendation
Khan et al. [21]	Belief	Affordability	Quicker service
Kuubiere et al. [22]	Belief	Affordability	Belief in faster healing
Moton et al. [23]	Belief	Affordability	Availability
Onyemaechi et al. [24]	Family and friend recommendation	Affordability	Belief
Panda and Rout [3]	Traditional skill and fame	Affordability	Availability
Safari et al. [14]	Affordability	Family and friend recommendation	Belief
Sayeed et al. [26]	Belief	Witchcraft	Family and friend recommendation
Solagberu [27]	Affordability	Belief in faster healing	Negative perspective of allopathic medicine
Udosen et al. [4]	Affordability	Belief in faster healing	Fear of Plaster of Paris/complications
Yempabe et al. [28]	Affordability	Belief	Quicker service
Zarrar et al. [29]	Affordability	Fear of surgery	Family and friend recommendation
Zehir et al. [30]	Avoiding long hospital treatment	Fear of complications	Discomfort in cast

**Table 3:** Patient-reported results by reason.

REASONS	NUMBER OF ARTICLES AS THE PRIMARY REASON	ARTICLES
Belief in TBS*	9	[1, 2, 11, 20–23, 26]
Affordability	8	[4, 8, 9, 13, 14, 27, 29]
Availability*	2	[1, 7]
Family and friend recommendation	2	[17, 24]
No choice	1	[10]
No improvement at allopathic site	1	[19]
Preference	1	[12]
Skill	1	[3]
Long treatment at medical clinic	1	[30]

*Hatipoglu and Tatar [1] had equal numbers of participants reporting availability and belief in TBS—as a result, they are counted in both categories.

### Qualitative patient perspectives

Of the 28 total articles, 5 included quotes from patients regarding their perspectives, experiences, and beliefs regarding TBS. As with the survey results, different themes emerged from the qualitative reporting. Positive perspectives praising TBS and reinforcing the belief in their practices were frequently reported, as were financial concerns [5, 6, 18, 25, 28]. Additionally, a single study reported negative perspectives of TBS, listing treatment failure and the limited ability of TBS to provide pain relief or antibiotics as reasons not to patronize TBS [18].

### Demographic association findings

The association between demographic trends and TBS usage was an area of further interest. Twenty-one articles included an analysis of whether patient demographics played a role in their decision to patronize TBS. While some articles found no association, others found conflicting associations. Jibo et al. and Zehir et al. found that TBS usage was associated with lower educational attainment, while Panda & Rout found the opposite association [3, 13, 30]. Three other studies found that occupation, socioeconomic status, or all demographic factors were associated with patient usage of TBS [10, 14, 17]. In contrast, 14 studies found no association between patient demographics and TBS usage, nor patients’ particular reasons for patronizing TBS [2, 4, 7, 9, 11, 19, 20, 22–25, 27–29].

## Discussion

In this study, two major themes representing the patients’ choice to patronize TBS became apparent: belief and preference, and accessibility and affordability.

### Belief and preference

We found that independent of accessibility, affordability, and demographics, patients often chose TBS over orthopedic practitioners based on a genuine belief in the practices and effectiveness of TBS. Given the longstanding tradition of TBS compared to the relatively new ideas of allopathic medical practices, Edusei et al. and Nwachukwu et al. both found that this tradition and the associated belief in TBS played a significant role in why patients chose them [5, 6]. This theme was found in many other articles, with belief in TBS being the primary reason cited in nine articles [2, 3, 11, 12, 20–23, 26].

Not only are patients expressing their belief in the practices and the spirituality of TBS’s care, but they are also highlighting the integration of TBS in their communities. In addition to “belief” as a primary reason, two articles found that recommendations from family and friends were the primary determinant in patronizing TBS. These reasons were also present in other articles, with five articles citing this as the second or third most common reason. Our results have shown that patients are actively choosing to patronize TBS based on belief in their practices as well as support in the community for these practices.

This theme highlights an important consideration. Patients are embedded in their communities and largely rely on the recommendations of family and friends for health care [17, 24]. The continuing presence of TBSs in their community, as well as a belief in both the spirituality and practicality of their practice, has been shown to influence patients’ decisions and can promote the patronage of TBS [5, 26, 27]. Exploring the nature of patients’ beliefs in TBS, including delineating between belief in practical applications and spirituality, is an important future direction. Moving forward, it is also necessary to understand TBS practices and how they are integrated into communities to fully understand why patients continue to patronize TBS, despite the complications.

A further contributing factor to patients’ preference for TBS was a history of negative experiences with allopathic medicine. Patients commonly highlighted a negative perspective of allopathic medicine and fear of orthopedic interventions (e.g., surgery, amputation, Plaster of Paris), which could act as another barrier preventing patients from seeking orthopedic care. Reflecting on the attitudes of health care professionals and the education surrounding orthopedic interventions may help to demonstrate why patients hold a negative perspective regarding allopathic medicine.

### Affordability and accessibility

As shown in previous articles, patients believe that TBS practices are cheaper than orthodox hospitals [7, 23]. The TBS centers are also thought to be more readily accessible [1, 7]. As a result, patients often choose the cheaper and more accessible treatment.

While different studies found disparate consensus on whether TBS was indeed more affordable, many patients believed that TBS was cheaper. This may contradict the actual costs in some areas. While Ariës et al. found that, on average, hospital treatments were €287 more expensive than TBS treatments, Dada et al. found the opposite, wherein hospital treatments were more affordable, with hospital treatments ranging from $34 to $98 and TBS prices ranging from $18 to $380 [9, 18]. Despite this discrepancy, the belief that TBS was cheaper was pervasive, which influenced many patients’ decision to patronize TBS [18]. Eight studies reported that affordability/cost was the primary reason for patronizing TBS [4, 8, 9, 13, 14, 27–29]. Other studies, including Ali et al. and Hatipoglu et al., found that availability was a primary reason to choose TBS, especially considering the barriers and delays experienced when accessing modern allopathic care [1, 7]. This highlights the importance of reducing health care costs and addressing delays in health care centers as a means of improving patient access to care. In the future, optimizing patient access to care may play a role in decreasing the overall morbidity and mortality of complications secondary to TBS practices.

### Demographic association

We also sought to understand whether there was a consistent association with patients’ demographics and their patronage of TBS, and if their demographics influenced their reasons for patronizing TBS. We found no consistent association of demographics and the use of TBS. The geographic distribution of the studies demonstrated a disparity in the amount of research on TBS done in different regions. While countries such as Nigeria and Ghana had many studies conducted across decades, other countries, including Bangladesh, India, Iran, and Uganda, had significantly less research published. This disparity suggests a need for further research on TBS in countries where research on the topic is less established.

### Limitations and strengths

The limitations of this study were primarily due to the quality of the included studies. Many of the articles collected had few participants, resulting in small sample sizes. Many studies used convenience sampling, recruiting their participants from hospitals and clinics, resulting in a biased sample of participants with a history of TBS complications. As a result, the participants included in the articles collected may not be representative of the population. Additionally, the results of the initial studies may have been biased by the perceptions of the original investigators. This study also only considered peer-reviewed articles written in the English language, excluding any relevant grey literature. Given the diversity of languages of the countries studied, relevant articles may have been excluded based on the language criteria. Additionally, while several articles highlighted belief in TBS, the nature of this belief (spiritual/other) was not specified. This may be the result of language barriers (lacking specific translations) or the lack of rigorous methodology in many studies that excluded the definition of terms. Nevertheless, this study still contributes to our understanding of TBS and patients’ perspectives on TBS usage.

## Conclusion

The reasons for patronizing TBS are multifaceted and extend beyond pure affordability and access to allopathic medical care. While affordability and access represent important reasons for patronizing TBS that must be addressed, additional considerations were demonstrated. This systematic review suggests that the general preference for TBS clinicians over allopathic practitioners and belief in the benefit of TBS practices is strongly rooted in individuals and communities that TBS serve. The contrasting negative perspective of allopathic medicine was stark.

Of the 28 articles, 27 suggested further public education campaigns on the harms of TBS, while other studies focused on improving the integration of TBS and modern orthopedic interventions to reduce complications. Increasing public education and collaborating with TBS practitioners to improve overall access to high-quality care, to deepen trust and belief in modern orthopedic practitioners, and to decrease complication rates may be future directions worth considering.

Future studies should seek to expand on the understanding of patients’ perspectives through quantitative means, as well as to pilot interventions promoting the integration of TBS and allopathic medicine.

## Additional File

The additional file for this article can be found as follows:

Supplementary Appendix 1. Example Search Strategy for Medline.
