# Supplementary material for: Patient Perspectives on Patronage of Traditional Bone Setting: A Systematic Review
Source: Ann Glob Health. 2026 Aug 31;92(1):86. doi: 10.5334/aogh.5295 (PMC13544037; doi:10.5334/aogh.5295)
Supplement: Supplementary file 1 [file agh-92-1-5295-s1.pdf]

Appendix 1: Example Search Strategy for Medline

Search History (4)

View Saved

#	Searches	Results	Type	Actions		Annotations
1	bone setting.mp.	134	Advanced	Display Results	More	
2	bone setters.mp.	97	Advanced	Display Results	More	
3	bonesetters.mp.	83	Advanced	Display Results	More	
4	1 or 2 or 3	283	Advanced	Display Results	More	

Save Remove Combine with: AND OR

Save All Edit Create RSS Create Auto-Alert View Saved Share Search History
